# Bio-inspired electronics: Soft, biohybrid, and “living” neural interfaces

**DOI:** 10.1038/s41467-025-57016-0

**Published:** 2025-02-21

**Authors:** Dimitris Boufidis, Raghav Garg, Eugenia Angelopoulos, D. Kacy Cullen, Flavia Vitale

**Affiliations:** 1 Department of Bioengineering, University of Pennsylvania, Philadelphia, Pennsylvania USA; 2 Center for Neurotrauma, Neurodegeneration & Restoration, Corporal Michael J. Crescenz VA Medical Center, Philadelphia, Pennsylvania USA; 3 Center for Neuroengineering and Therapeutics, University of Pennsylvania, Philadelphia, Pennsylvania USA; 4 Department of Neurology, University of Pennsylvania, Philadelphia, Pennsylvania USA; 5 Department of Neurosurgery, University of Pennsylvania, Philadelphia, Pennsylvania USA

**Keywords:** Biomedical engineering, Bioinspired materials, Regeneration and repair in the nervous system, Bionanoelectronics

## Abstract

Neural interface technologies are increasingly evolving towards bio-inspired approaches to enhance integration and long-term functionality. Recent strategies merge soft materials with tissue engineering to realize biologically-active and/or cell-containing living layers at the tissue-device interface that enable seamless biointegration and novel cell-mediated therapeutic opportunities. This review maps the field of bio-inspired electronics and discusses key recent developments in tissue-like and regenerative bioelectronics, from soft biomaterials and surface-functionalized bioactive coatings to cell-containing ‘biohybrid’ and ‘all-living’ interfaces. We define and contextualize key terminology in this emerging field and highlight how biological and living components can bridge the gap to clinical translation.

## Abstract (web summary)

Neural technologies are adopting bio-inspired designs to enhance biointegration and functionality. This review maps the growing field of bio-inspired electronics and discusses recent developments in tissue-like bioelectronics, from soft interfaces to ‘biohybrid’ and ‘all-living’ platforms.

## Introduction

**Fig. 1:**
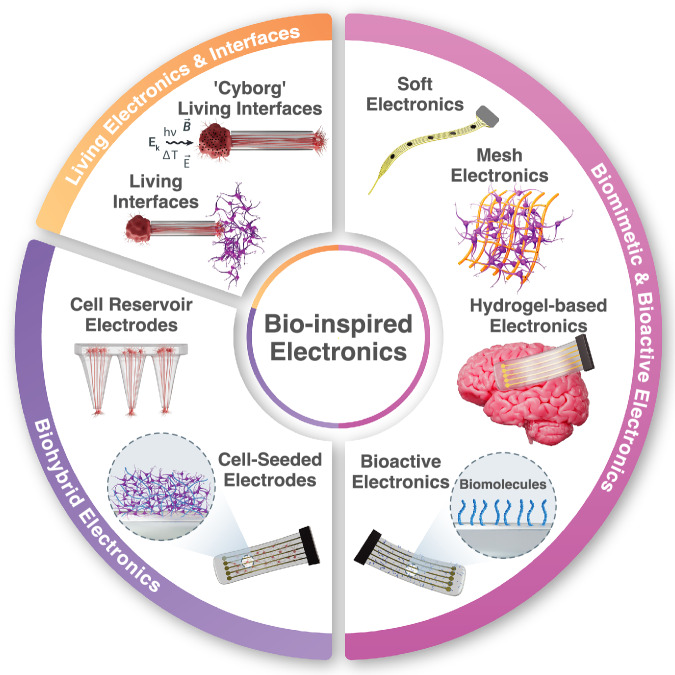
Bio-inspired Electronics. Schematic overview of emerging strategies for bio-inspired electronics and neural interfaces.

The rapid rise of neuroelectronics is changing clinical diagnosis and management of various disorders by introducing novel invasive and wearable technologies able to precisely monitor and modulate physiological functions at the cell, organ, and circuit level^1–5^. Non-invasive brain mapping techniques, such as scalp electroencephalography (EEG), are essential in the diagnosis and monitoring of neurological diseases such as epilepsy, sleep disorders, Parkinson’s, stroke, brain tumors, and more^6–11^. EEG is widely adopted in clinical practice due to its low cost, safety, and ease of deployment, even if recordings are limited to low-frequency activity generated in the underlying cortical regions^12^. In contrast, invasive technologies such as brain-computer (BCI) and brain-machine (BMI) interfaces allow high-bandwidth recordings from deeper brain structures, including both intracortical and subcortical targets^5,13,14^. An early demonstration of invasive interfaces implanted into a human participant was first reported in 1998^15^, followed by successful demonstrations of human BCIs with Utah microelectrode arrays (MEAs) in the first BrainGate trials in the early 2000s^16,17^. Over the past few decades, advances in implantable electronics have resulted in new knowledge on brain function, disease, and behavior, which have, in turn, enabled and advanced novel therapeutic strategies. Some examples include electrocorticography (ECoG) and stereo EEG for presurgical and intraoperative epilepsy monitoring^18–20^, responsive neurostimulation^21,22^, speech decoding^23–25^, and motor recovery following spinal cord injury^26^, closed-loop sensory-motor interfaces for prosthetic control^27,28^, as well as deep brain stimulation (DBS) for Parkinson’s Disease^29,30^ and neuropsychiatric conditions^14,31^. Today, the emerging field of neuroelectronics attracts increasing attention and support from academia, government, and industry, with many of these technologies already advancing through the translational pipeline toward clinical use^32^. Despite such remarkable progress, the fundamental mismatch between the properties of man-made electronics and biological substrates still profoundly limits the functionality, safety, and lifetime of neuroelectronic implants. In this review article, we chart the emerging strategies that have been proposed for the development of bio-inspired electronics and interfaces, ranging from biomimetic tissue-like electronics to biohybrid and all-living approaches, ultimately aimed at the seamless structural and functional integration between implants and host tissues (Fig. 1).

Traditionally, neural interfaces for human and animal use, such as DBS leads, Utah arrays, Michigan-style laminar probes, and Neuropixel, rely on rigid conducting and semiconducting materials, such as platinum (Pt) and its alloys, gold (Au), and silicon (Si)^33^. Advances in microfabrication have pushed the limits of electrode number and density, but significant challenges such as inflammatory response to implants, functionality, and material degradation over time remain unaddressed^34^. A key determinant of the quality and longevity of the electrode-tissue interface is the mechanical mismatch between rigid materials and much softer neural tissue (e.g., Si ~ 180 GPa, brain ~ 1–30 kPa)^35,36^. Such pronounced mechanical and structural mismatch prevents rigid devices from conforming to biological substrates, introduces signal instability, and results in physical damage to neural tissue during insertion as well as from tissue micromotion during indwelling^34^. Initiated immediately upon implantation, the host organism responds to local trauma and identifies the devices as foreign entities, triggering an inflammatory response^37^. Rigid materials exacerbate the foreign body response (FBR) and the formation of a glial scar encapsulation that leads to gradual signal degradation, decline of recording and stimulation capabilities, and increase in electrode impedance^38–41^. In addition, continuous tissue micromotion, pulsation, and friction against rigid electrodes significantly contribute to the severity of the FBR^34^. Finally, rigid electrodes cannot accommodate tissue displacement, as well as volumetric and density changes occurring during development, aging, and disease^34^.

To overcome these challenges and seamlessly integrate neuroelectronic devices with host brain structures, design strategies and material selection have been increasingly directed toward the development of biomimetic electronics that are increasingly tissue-like. Soft and flexible devices – engineered to better match the mechanical properties of biological tissues – modulate FBR by minimizing mechanical trauma^42^ and reducing micromotion-induced damage^43,44^. Solutions to enhance device flexibility involve a combination of design-based approaches, such as mesh structures^45–47^, fibers^43,48–51^, and ultra-thin films^52–55^, and engineered material choices, such as soft polymers and elastomers^56,57^, hydrogels^3,58,59^, and conductive nanocomposites^60,61^. While minimizing device footprint and thickness can somewhat mitigate the mechanical mismatch with the tissue^62,63^, a growing body of literature suggests that materials with elastic moduli and compositions closely resembling those of neural tissues can further reduce FBR and achieve long-term, stable integration^48,61,64,65^. Furthermore, functionalizing the surface of electronic components with biomolecules could harness biochemical cues from the host tissue microenvironment and the extracellular matrix (ECM)^65^, thus realizing ‘bioactive’﻿ electronics. In ‘biohybrid’ neural interfaces a layer of living cells at the brain-device interface serves not only to better emulate native tissues but can also act as an active scaffold to promote tissue regeneration, cell migration, and differentiation, while also monitoring these processes by transducing bioelectronic signals^66–69^. Finally, ‘all-living’ approaches for synaptic-mediated control of neural circuits further underscore a new paradigm in bio-inspired electronics that are solely composed of biological components and living cells^70^. The key terminology that underpins this emerging field is herein defined and contextualized, organizing key research developments and major challenges into discrete subsections of the bio-inspired electronics continuum (Box 1).

BOX 1 Navigating the terminology of bio-inspired electronics

- Biomimetic electronics are engineered to mimic tissue mechanics, enhancing compatibility and reducing trauma by using soft, flexible designs, and organic or synthetic materials like polymers or hydrogels to reduce stiffness and better match the mechanical properties of biological tissues.
- Bioactive electronics integrate biologically-derived components such as extracellular matrix (ECM) proteins, growth factors, and/or adhesion molecules, which interact with their surrounding environment to promote cell proliferation and tissue regeneration. Bioactive electronics can be further functionalized for cell-specific targeting, minimal microglia recruitment, and even provide on-demand drug and gene delivery.
- Biohybrid electronics contain living cells that create a biological layer at the device/tissue interface, improving biointegration and potentially acting as active scaffolds for probing pathophysiology and/or promoting tissue regeneration.
- Living electronics and interfaces are only composed of biological components and living cells that function as active input/output components of the device. Information exchange between the implant and host tissues is primarily recorded, transduced, and modulated by living cells instead of synthetic components.

## Biomimetic and bioactive electronics

### Biomimetic electronics

**Fig. 2:**
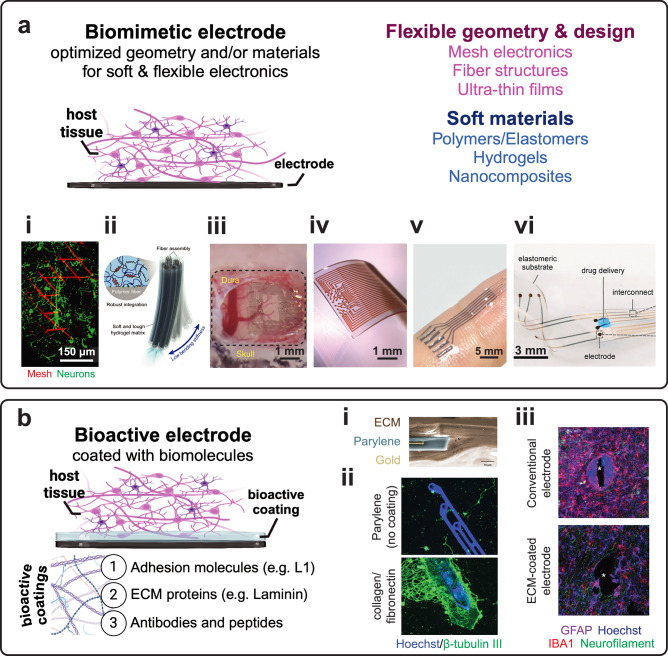
Biomimetic and bioactive electronics. **a** Schematic of biomimetic electrodes. Materials and designs are optimized for soft and flexible electronics. **i**. Interpenetration between neurons (β-tubulin; green) and mesh electronics (red) after co-injected into Matrigel for 14 days^45^. **ii**. Illustration of flexible hydrogel probe design with multifunctional fiber units^48^. **iii**. Ultra-thin electrode array for long-term recordings from the rat cortical surface^53^. **iv**. Stretchable, high-density grid of Au-coated titanium dioxide nanowire electrodes in a silicone matrix^56^. **v**. All-hydrogel bioelectronic interface based on a bi-continuous conducting polymer hydrogel^59^. **vi**. Electronic dura mater (*e-dura*) with stretchable Au interconnects, soft Pt/silicone electrodes, and microfluidic drug delivery channel to restore locomotion in paralyzed rats^60^. **b** Schematic of bioactive electrodes coated with biomolecules. **i**. SEM image of the cross-section of ECM-coated Au-parylene C microelectrode array^65^. **ii**. Confocal fluorescent images showing neurite outgrowth, network formation (β-tubulin III; green), and neuronal nuclei presence (Hoechst; blue) for non-coated and collagen I/fibronectin-coated Au-parylene C neural electrodes^221^. **iii**. Confocal fluorescent images showing reduced response of ECM-coated Au-parylene C microelectrodes at 2 mm below the cortical surface compared to silicon microelectrodes (GFAP – astrocytes: lilac; Iba1 – microglia: red; neurofilament – neuronal axons: green, Hoechst – nuclei: blue)^221^. Panels reproduced with permission from (**a**)**. i**. ref. ^45^., Nature; **ii**. ref. ^48^., Nature; **iii**. ref. ^53^., Nature; **iv**. ref. ^56^., Wiley; **v**. ref. ^59^. Nature; and **vi**. ref. ^60^. Science; and (**b**)**. i**. ref. ^65^., PLOS; **ii**. ref. ^221^., Nature; and **iii**. ref. ^221^., Nature. Panels (**a** and **b**) created with BioRender.com and released under a CC BY-NC-ND 4.0 International license (creativecommons.org/licenses/by-nc-nd/4.0/deed.en).

Biomimetic neural interfaces and electronics mimic the physical properties of the target tissues for static structural integration, by optimizing the design and/or material selection to reduce inflammation and FBR^47,52^, minimize strain from implant micromotion^71^, and seamlessly conform to the morphological and biochemical properties of tissues^60^ (Fig. 2a). Biomimetic electronics can be broadly classified based on geometric and design principles (e.g., ultra-thin metallic or semiconducting structures to minimize flexural rigidity^52–55^, open-faced and three-dimensional (3D) mesh geometries to enhance integration with the host tissue^45–47^, serpentine structures for stretchability^72^, etc.), or based on the constituent materials (e.g., soft polymers^53,56,57,73^, hydrogels^3,58,59^, low-density nanomaterials^51,54,55^, and nanocomposites^60,61^). After years of preclinical development and validation, biomimetic neural interfaces relying on micro-scale electrodes, such as Synchron’s stentrode^74^, Neuralink’s threads^75^, and Precision Neuroscience’s thin-film microECoG grids^76^, are now advancing in clinical trials toward commercialization, and many more are in the pipeline^17,32^.

#### Polymer-based electronics

Polymers offer a combination of flexibility, inertness, electrochemical stability, and durability, which are essential for long-lasting, tissue-conforming electronics^33^. Polymer-based substrates and coatings have been proposed to reduce the mechanical mismatch at the electrode-tissue interface, while conductive polymers have been developed to reduce electrode impedance^77^. Insulating polymers like polydimethylsiloxane (PDMS), parylene-C, SU-8, polyimide (PI), and polyethylene terephthalate (PET) possess the required inertness, medium-term hermeticity, biocompatibility, and processibility with conventional lithographic and micromachining techniques^33^. As such, these polymers are the typical materials of choice for the substrate and encapsulation of flexible neuroelectronic interfaces^78^. For example, a PDMS-based implant, comprised of a PDMS substrate, Au interconnects, and soft electrodes with a Pt-PDMS composite, was developed to mimic the shape and elastic modulus of the spinal dura mater^60^. The implant – named the *e-dura* – was used to restore locomotion after spinal cord injury in rats by concurrent serotonergic drug delivery through a microfluidic channel and continuous electrical stimulation to specific spinal segments^60^. No significant difference was observed in the expression of activated astrocytes or microglia in lumbosacral spinal cord sections of rats implanted with the e-dura for 6 weeks compared to sham^60^. Similarly, direct photolithography of Au on SU-8 was used to fabricate endovascular probes that can be delivered into sub-100 μm vessels of rat brains^79^. Once injected, these flexible probes adhere like a stent to the walls of the blood vessel and can be used to record local field potentials as well as single-unit (SU) spikes with minimal chronic inflammatory response^79^. The same polymer has also been used for ultrathin (<1 μm) neuron-like Pt electrodes, engineered to approximate the mechanical properties of neural cells^49^. These probes exhibited bending stiffness of approximately $1.4 \sim 5.7\times {10}^{-16}{\rm{N}}\cdot{{\rm{m}}}^{2},$ which was at least 5 times lower than that of other flexible neural implants^63,80–82^ and comparable to that of an axon ($5.9 \sim 7.6\times {10}^{-16}{\rm{N}}\cdot{{\rm{m}}}^{2}$, depending on the diameter)^49,83^. Thin-film microelectrode arrays have also been fabricated by micropatterning nanomaterials like graphene^55,84–87^, Ti_3_C_2_T_x_ MXene^54,88,89^, carbon nanotubes^90^ and Pt nanorods^91^ on thin (<10 μm) polymeric substrates.

Unlike traditional polymers, which are typically insulators, conductive polymers can conduct electric current due to their unique conjugated molecular system^92,93^, and have been investigated for neuroelectronic applications due to their flexibility and electrochemical stability^33,94,95^. Poly(3,4-ethylene-dioxythiophene) polystyrene sulfonate (PEDOT:PSS) has been the most extensively used conductive polymer, both in the form of coatings as well as free-standing films to reduce impedance and enhance the signal transduction capabilities of neural electrodes^93,95^. For instance, NeuroGrid, an ultrathin (4 μm) electrode array with free-standing PEDOT:PSS flexible electrodes, has been successfully used to detect single-cell action potentials from the surface of the rat brain for up to 10 days^53^, as well as in active neuroelectronics (i.e., with on-board signal amplification) based on PEDOT:PSS organic electrochemical transistors (OECTs)^96–99^. Furthermore, PEDOT and polypyrrole (PPy) nanotubes on iridium (Ir)-based Michigan electrodes have been shown to not only enhance the electrochemical properties of the electrodes but also to promote neurite outgrowth in dorsal root ganglia explants compared to uncoated Ir implants^100^. All-polymer soft electronics composed of an inner PDMS-PEG-PEDOT core and an outer insulating layer of fluorosilicone or parylene C, have been shown to reduce microglia attachment and improve neuronal adhesion compared to stiff controls in vitro^43^. Acute in vivo testing showed that the fluorosilicone-coated soft electrodes could record evoked action potentials in the adult rat visual cortex^43^.

Engineering specific morphological and topological features like macro-, micro- and nano-porosity, as well as mesh-like geometries to achieve structural flexibility and bio-integration^45,101^ is another approach that has been successfully adopted to minimize tissue trauma and scarring. Specifically, the open structure mesh-based implants result in a reduction in the total implant footprint flexural rigidity^102^ and may favor tissue ingrowth^101^ and nutrient diffusion^62^. For instance, mesh electrodes (<1 μm) composed of SU-8 and Cr/Au layers exhibit four orders of magnitude smaller bending stiffness compared to thin PI probes (25 μm, mesh: 0.104 nN⋅m, PI: 3.3 × 103 nN⋅m) and do not cause long-term changes in neuron and glia distribution at the mesh-tissue interface at 3 months post-implantation in mouse brains^101^. Chronic in vivo recording and stimulation studies demonstrated stable local field potentials and unit recordings in mouse brains for at least 8 months^80^. A different variation of mesh arrays used bioresorbable silk fibroin to facilitate the fabrication of ultra-thin (2.5 μm) electronics^52^. These arrays were tested in the feline visual cortex and sleep spindles with high amplitude and signal-to-noise ratio (SNR) were detected over an implantation period of 4 weeks^52^. Moreover, stretchable mesh nanoelectronics have been developed to achieve long-term and stable electrophysiological measurements of developing brain organoids^103^ and single-cell-level recording of the same neurons over the entire adult life of mice^47^.

#### Hydrogel-based electronics

Hydrogels are 3D networks of crosslinked organic and inorganic materials that can absorb and retain significant amounts of water^104,105^. Traditionally, hydrogels are composed of polymeric molecules; however, numerous polymer-free hydrogels have recently been developed^106,107^. Depending on the composition or specific modifications, hydrogels can be insulating or conductive via the integration of ionic liquids and electrolytes, nanomaterials, or conductive polymers, which makes them suitable for use as both passivation layers and electrode contacts^3^. Due to the high water content, hydrogels are inherently soft and can match Young’s modulus of host neural tissues, significantly mitigating the stiffness-induced FBR^3^. Furthermore, their versatility and tunable electrical, mechanical, and chemical properties are instrumental in bridging the gap between rigid electronics and the dynamic, soft, and organic nature of biological tissues.

Soft hydrogel coatings like polyethylene glycol (PEG) and poly(vinyl alcohol) (PVA) on rigid electrode structures have been shown to effectively reduce glial scar formation and neuronal loss^3,71^. Polyethylene glycol dimethacrylate (PEG-DMA) hydrogel coatings on borosilicate glass capillaries were found to be effective in mitigating the frictional forces from tissue-implant micromotion, and subsequently reducing the gliotic scarring from strain fields around the implant^71^. Metamaterials with novel functionalities can also be synthesized by incorporating nanomaterials into a hydrogel matrix, resulting in soft, stretchable, and electrically conductive hydrogel composites^108^. For instance, viscoelastic alginate matrices have been combined with Ag flakes^109,110^, and Ag-polyacrylamide-alginate hydrogel composites were used to develop skin-mounted electrodes for neuromuscular electrical stimulation^110^. A hydrogel-based fully viscoelastic array was fabricated using an ionically conductive alginate matrix enhanced with graphene flakes and carbon nanotubes, with minimal activation of astrocytes and enhanced neurite spreading in vitro^58^. This array was validated in vivo via muscle stimulation in mice, ECG recordings in mouse hearts, and low-amplitude local field potentials from the epidural surface of rat cortex. Highly conductive (867 S m^–1^) PPy-PEDOT:PSS hybrid hydrogels with a hierarchical porous structure enhanced PC12 cell viability and realized highly sensitive electrochemical biosensing of dopamine in vitro^111^. Conductive polymer (polyaniline, PPy, or poly-aminoindole) hydrogels have also been crosslinked with PEDOT:PSS as a conductive dopant, with improved viability in vitro and the ability for in situ sensing of bioactive molecules (e.g., dopamine and hydrogen peroxide) released from living cells^112^. Furthermore, monolithic 3D-printed all-hydrogel bioelectronic interfaces were shown to effectively stimulate and record the electrophysiological activity of several rat tissues and organs in vivo and stimulation of rat sciatic nerves and spinal cords^59^.

### Bioactive electronics

Neural electrode implants can be decorated with bioactive components that match or resemble the biochemical milieu of the host tissue to enhance cell adhesion^113^, evade the immune response^114^, and minimize glial scar formation^115^ (Fig. 2b). Bioactive coatings often include extracellular matrix (ECM) proteins, adhesion molecules, and growth factors to promote long-term biocompatibility and attract neurite ingrowth, as well as antibodies that harness interactions between cells and the surrounding microenvironment for cell-type specific targeting of neural probes^116–118^.

To minimize inflammatory immune response and glial scar encapsulation, Si-based laminar MEA probes were treated with the neuronal cell adhesion molecule L1 and showed a greater acute reduction in microglial surface coverage and activation of distant microglia compared to untreated probes in vivo^114^. Over 16 weeks, L1-coated Si arrays implanted in the primary visual cortex of mice showed a higher yield of visually evoked SUs, higher SU amplitude, and SNR, while increasing neuronal density and decreasing microglial activation compared to bare Si implants^119^. Another study examined whether the addition of a laminin coating would reduce the glial response to Si MEAs. Despite an increase in microglia activation 1-day post-implant, indicating a potential acute stimulatory effect of laminin on microglia, a long-term reduction of the glial scar was observed in a rat model at 1 month in vivo^115^. Similarly, coating Si MEAs with an astrocyte-derived mixture of ECM proteins led to decreased glial scar formation compared to U.S. Food and Drug Administration (FDA) approved collagen-based coatings^118^. Another biomolecule, hyaluronic acid (HA), was combined with PPy and deployed as a coating on Ir microwires, significantly reducing glial scarring after three weeks in vivo^120^. Coatings primarily composed of ECM proteins were shown to not alter the impedance and mechanical properties of microfabricated Au/parylene C microECoG arrays and were effective in reducing glial scarring at 7 and 30 days after subdural implantation in rat somatosensory cortex compared to uncoated arrays^65^. In addition to serving as neuroprotective coatings, neural cell adhesion molecules or ECM proteins integrated in hydrogels can realize bioactive electronics with active drug and molecule delivery functionalities, including nerve growth factors^121^ and anti-inflammatory drugs^122^, such as dexamethasone^123^ and α-MSH^124^.

To enhance cell adhesion and differentiation, a layer-by-layer assembly approach was utilized to coat a Si/Si${\text{O}}_{2}$ substrate with alternating nanometer-scale films of polyethyleneimine (PEI) or chitosan with either gelatin or laminin. PEI-laminin multilayers showed the best adhesion to cortical neurons and remained stable for at least 7 days in vitro in simulated physiological conditions, while not affecting the MEA impedance^113^. Similarly, PPy substrates doped with the ECM molecule chondroitin sulfate and functionalized with type I collagen were shown to promote PC12 cell differentiation and neurite outgrowth^125^. In another study, carboxy-endcapped polypyrrole (PPy-α-COOH) films modified with a common cell-adhesive motif (arginylglycylaspartic acid) had a higher cell adhesion and spreading compared to unmodified PPy-α-COOH films and standard PPy films, without altering the film conductivity^126^. Bioactive coatings may also be used for cell-specific targeting. For instance, SU-8-based mesh electronics functionalized with antibodies (anti-EAAT2, anti-CD11b, anti-D2DR) and a laminin-1-derived synthetic peptide, when implanted in vivo in the mouse hippocampus, allowed specific targeting of cell types (neurons, astrocytes, and microglia) and even neuron subtypes (D2R-expressing neurons) in chronic electrophysiological recordings and longitudinal histological analysis^116^. Cell membrane-mimicking conducting polymers based on ethylene-dioxythiophene have also been proposed. In those conducting polymers both biochemical (with laminin-1-derived synthetic peptide conjugation) and electrical stimulation capabilities were integrated to achieve selective binding of PC12 cells and enhanced neurite outgrowth^117^.

### Challenges and outlook

The reliance of biomimetic platforms on thin-film conductors and conductive polymers allows these interfaces to exhibit low electrode impedance owing to their intrinsically high electrical conductivities and electrochemical capacitances^87,127,128^. As a result, electrophysiological recordings with a high signal-to-noise ratio are possible since the magnitude of thermal noise in the recordings is directly proportional to the electrode impedance^129,130^. Interfaces with high electrochemical capacitance also enable efficient electrical stimulation while minimizing unwanted and potentially harmful irreversible faradaic reactions at the electrode-tissue interface^127,131^. These characteristics make biomimetic platforms favorable for electrophysiological recording and stimulation. The electrical conductivity of hydrogel-based electronics depends on the percolative network of the conductive fillers within the bulk of the hydrogels, the intrinsic electrical conductivity of polymeric chains, and the ionic mobility through the bulk water^132,133^. This structural composition does not facilitate conductivities as high as metals and nanocarbons. However, the mechanical compliance of hydrogels with biological tissues enables safer chronic applications. In the case of bioactive interfaces, the structural and electrical properties of the bioactive species govern the overall functional properties of the interface. For example, adhesion-promoting coatings will tighten the coupling with target neurons, resulting in improved quality of recordings. Given the compatibility of biomimetic and bioactive interfaces with existing data acquisition and stimulation systems, these interfaces are well suited for electrophysiological recordings with high transfer bandwidths and stimulation capacity.

The long-term challenges of neural interfaces include ensuring device stability and performance in chronic physiological conditions, mitigating foreign body response and glial scar formation, and addressing manufacturing scalability. Biomimetic devices depend heavily on the durability of electrode materials, which must endure physiological stresses over time for consistent performance. Bioactive electronics face additional challenges such as chronic efficacy, safety, bioavailability, controlled biomolecule release, mitigating inflammation from enzymatic degradation, and navigating complex regulatory pathways^94,134,135^. Future directions should focus on developing biomimetic and bioactive electronics with properties that adapt to changes in the tissue environment, including different stages of tissue growth, development, and post-implantation healing. Incorporating dynamic functionalities in the polymeric substrates through shape-morphing materials and topographical bioelectronics offers promising avenues for realizing such platforms. Shape-morphing devices can dynamically adapt their form to enhance tissue conformity and signal fidelity in stimulation and recording^136–139^, while topographical electrodes leverage surface patterning to guide cellular alignment, facilitating improved electrode-tissue integration^134,140^. In addition, bioactive electronics could evolve into smart biointerfaces that can actively sense biochemical cues and respond with targeted, on-demand drug delivery or electrical stimulation. Given the challenges of chronic stability in neural interfaces, there is also a growing interest in biodegradable or transient electronics for applications that do not require long-term functionality^141,142^.

## Biohybrid electronics

**Fig. 3:**
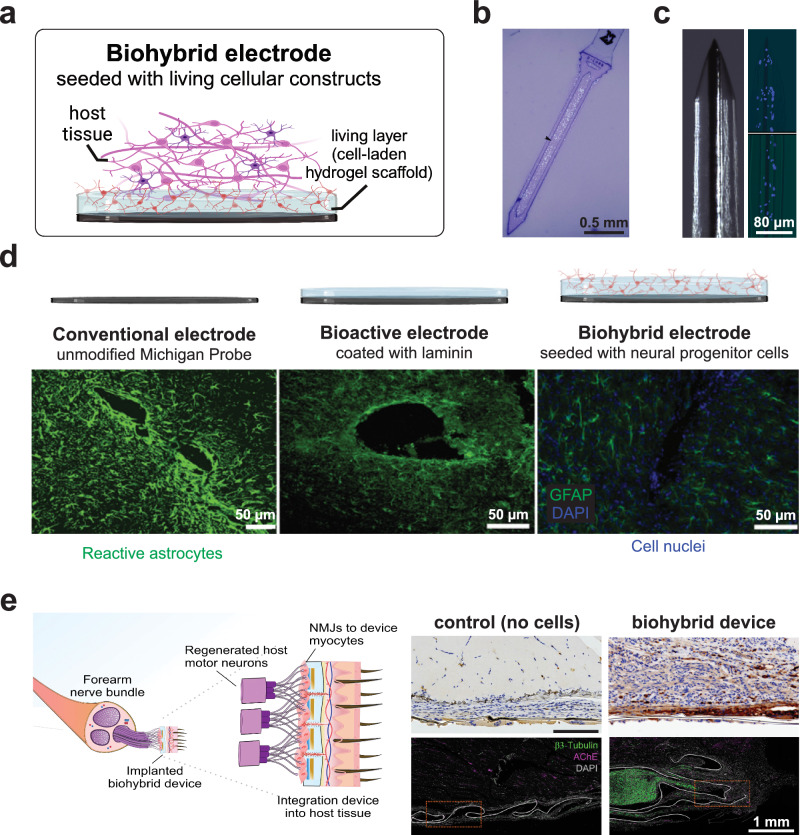
Biohybrid electronics. **a** Schematic of a biohybrid electrode, seeded with living cells. A cell-containing living layer serves as a biological interface between synthetic electronic components and the host tissue. **b** Neural stem cell-seeded probe (Hoechst staining nuclei; blue)^148^. **c** Microelectrode with a cell-laden biodegradable fibrin hydrogel coating (DAPI staining nuclei; blue)^164^. **d** Reduced glial scar of silicon-based electrodes with neural progenitor cells grown on a laminin coating^149^. Reactive astrocytes are stained with GFAP (green) and cell nuclei with DAPI (blue). **e** Flexible biohybrid device seeded with myocytes forms neuromuscular junctions (AChE; pink) for functional nerve restoration after injury^168^. No NMJs are observed in control devices without cells. Panels reproduced with permission from (**b**). ref. ^148^., IOP; (**c**). ref. ^164^., Frontiers; (**d**). ref. ^149^., JNSPG; and (**e**). ref. ^168^., Science. Panels (**a** and **d**) (top) created with BioRender.com and released under a CC BY-NC-ND 4.0 International license (creativecommons.org/licenses/by-nc-nd/4.0/deed.en).

Merging tissue engineering approaches with bioelectronics is a promising route to improve the biocompatibility and long-term integration of neural interfaces by engineering a biological platform within the device for integration with host cells^67,68^. Traditionally, biohybrid electronics research has focused on seeding living cells directly on electronic devices or encasing the cells into cell-laden hydrogel scaffolds (Fig. 3a). One of the first attempts to merge conventional electrodes with cells was the neurotrophic “cone electrode” (1988)^143^. A hollow glass cone housing insulated gold wires and enclosing a fragment of the sciatic nerve was used to promote cortical neurite ingrowth into the cone and onto the recording surface, yielding stable recordings for up to 15 months^143^. Interestingly, attempts to replace the living biological component (i.e., sciatic nerve) with biomolecules (i.e., neurite growth factors) resulted in reduced neurite ingrowth, thus highlighting the benefits of cell-containing systems that extend beyond mere bioactive coatings on a synthetic surface. This strategy transitioned to human clinical trials, demonstrating over a decade of stable recordings in a locked-in patient^144^. Remarkably, histological analysis 13 years post-implantation showed neurite growth into the electrode tip without signs of glial scar, demonstrating the integrative capability of a biohybrid neuroelectronic interface^144^. Another initial example of a biohybrid neural interface is the sieve electrode with a cell container developed in 2002 to interface with peripheral nerves after traumatic lesions^145^. Implanted on the distal end of the nerve stump, this ‘neuron microprobe’ was the first biohybrid device with microsieve ring electrodes that contact axon projections growing across a cell container. Axons in the biohybrid device acted as mediators for chronic coupling between the microelectrodes and the target muscles, to preserve neuromuscular junctions and restore skeletal muscle control after peripheral nerve injury^145^. Since then, several biohybrid strategies have been proposed to incorporate living cells into implantable devices, including the attachment of cells to electrodes functionalized with ECM-derived biomolecules and cell-laden hydrogel scaffolding for functional nerve restoration^38,66–68^. Tissue-engineered neural-electrical relays have also been developed by growing neurons directly on electrically conductive polymer fibers and subsequently coated using a thin agarose hydrogel layer to maintain neuronal network adhesion on the fibers^146^. Small-diameter (<400 μm) polyaniline–polypropylene (PA-PP) fibers were coated with collagen and supported primary dorsal root ganglion (DRG) neuron adhesion and neurite outgrowth, representing a promising approach to building arrays of mechanically compliant electrodes pre-seeded with living neuronal networks^146^. Similarly, a neuroprosthetic interface using stretch-grown engineered axonal tracts plated on PI-based flexible MEAs was developed to interface MEAs with regenerating peripheral nerves. The axon/MEA assemblies were grown in vitro, embedded in an agarose matrix, inserted into 4 mm nerve guidance tubes, and sutured to transected sciatic nerves, showing host axonal ingrowth and vascularization as early as 2 weeks in vivo^147^.

### Cell-seeded electrodes to improve the device-tissue interface

While it was recognized early on that living cells could affect the microenvironment of implanted electrodes, it was not until recently that researchers systematically assessed how biohybrid devices might influence surrounding neural cell density^148^. Parylene C-based arrays fabricated with a hollow well to contain neural stem cells within an alginate hydrogel (Fig. 3b) were shown to support host neuronal survival and reduce the tissue response for 1-month post-implantation, mainly through secreted neuroprotective factors^148^. However, at later time points, degradation of the hydrogel encapsulation led to reduced neuronal viability in the vicinity of the implant, showing the importance of tuning the mechanical and biochemical properties of the hydrogels to promote and ensure cell survival^148^. With a similar strategy, neural progenitor cells grown on laminin-coated silicon-based electrodes exhibited improved integration and reduced glial scarring, with neurotrophic factors being released by astrocytes around the implant (Fig. 3d) for up to a week^149^. However, chronic performance beyond a few weeks in vivo is necessary to advance such technologies toward clinical use.

An alternative approach based on a neural spheroid cultured in a microchamber at the tip of a penetrating electrode has been conceptualized for neural stimulation deep in brain structures^150^. However, relying on unguided axonal growth from the spheroid might limit the practicality of such an approach, and this has only been tested in vitro. Similarly, a parylene C-based flexible MEA with an array of neurospheroids was used to activate 2D cortical neuron cultures in vitro^151^. A different approach involving flexible and transparent silk films with micropatterned electrodes has also been reported^152^, where microgrooves regulated glial cell alignment and guided spatially confined growth of cortical neurons. Here, the interface functionality was validated in vitro by measuring the Ca^2+^ response upon electrical stimulation of cortical neurons. Another strategy to interface living cells with electronic materials relies on the polymerization of PEDOT on electrodes seeded with neural cells in vitro^153^. The conductive polymer wrapped around the neuronal somas and axons, and electrochemical characterization revealed a distinct contribution of living cells in the PEDOT matrix. Although viability was maintained for almost 1 week, apoptosis of neurons trapped within the PEDOT matrix was then observed, possibly due to physical and biochemical disruption of the integrin signaling, lack of cellular adhesion with ECM proteins, and oxidative stress by cytoskeletal changes^154,155^.

The incorporation of cells into hydrogel substrates offers numerous advantages, including mechanical compliance, cell protection, and an ECM-like microenvironment, which ultimately supports graft cell survival and long-term functionality of the electrode surface. Hydrogels are extremely versatile platforms with tunable mechanical and chemical properties^105^, that create a microenvironment conducive to cellular growth and viability within three-dimensional cultures. Biochemical signaling cues and properties such as mechanical stiffness, degradability, and viscoelasticity directly influence key cellular processes, including cell fate determination, differentiation, proliferation, adhesion, and spreading, as well as cell-cell and cell-matrix interactions^156–160^. Dynamic tuning of these characteristics also allows hydrogels to model physiological changes observed in aging and neurodegenerative diseases, providing critical insights into how altered biophysical cues affect cellular functions and behavior^158,161^. With over 100 hydrogel products approved by the FDA and European Medicines Agency (EMA)^162^, and a growing number of clinical trials exploring novel hydrogel biomaterials for emerging applications, hydrogels have demonstrated significant success as biomaterials in both preclinical and clinical settings^162,163^. Polymers like hyaluronic acid (HA), silicone, poly(ethylene glycol) (PEG), collagen, and cellulose represent over half of the approved hydrogel products and account for the majority of hydrogels currently in clinical trials^162^. To improve the chronic performance of intracortical implants, hybrid microelectrodes were seeded with hippocampal neurons or astrocytes and encased for protection by a thin biodegradable fibrin hydrogel coating (Fig. 3c)^164^. Notably, the hydrogel layer housing living cells reduced reactive astrocytes without significantly altering the electrode impedance. Moreover, the complete reabsorption of the fibrin hydrogel within 7 days may overcome the issue of the hydrogel swelling in vivo with the consequent increase of the distance between the electrodes and the host cells^164^. As a notable development in the field, biohybrid devices consisting of a hydrogel bilayer structure with a biodegradable cell-laden layer on top of a conductive hydrogel have been reported^165,166^. In these multilayer structures, the biodegradable hydrogel addressed the viability loss of neural progenitor cells contained within and further reduced the mechanical mismatch between the tissue and the electrode, while the conductive hydrogel layer at the surface of the metal electrodes improved the charge storage capacity and injection limits compared to untreated Pt devices^165,166^. However, limited neurite outgrowth and no synapse formation were observed, further showing the need for motivation and guidance of outgrowth post-implantation^165^. Despite the advantages, the issues of host tissue response, glial scar formation, and graft rejection of cell-laden hydrogels still need to be addressed. Moreover, directed growth and migration of cells post-implantation must be controlled to ensure integration, minimize cell loss, and eliminate the risk of aberrant growth. Lastly, while most studies focus on the effects of the hydrogel properties on neurons, the physical and biochemical environments provided by the hydrogel are also crucial for glial cells. Indeed, the stiffness of a PVA hydrogel enhanced with sericin and gelatin (PVA-SG) plays a significant role in glial cell morphology and ECM protein deposition, which are essential in the development of functional neural tissues^167^.

### Cell-seeded electrodes for functional restoration

#### Regenerative bioelectronics for functional nerve restoration

Recent advances in biohybrid regenerative bioelectronics have facilitated the functional restoration of peripheral nerves post-trauma and amputation^168^. A parylene-C device with Au tracts and PEDOT:PSS microelectrodes seeded with induced pluripotent stem cell (iPSC)-derived human skeletal myocytes in a fibrin hydrogel formed mature myofibers by 8 days in vitro (Fig. 3e)^168^. Post-implantation, this biohybrid device formed neuromuscular junctions as evidenced by the acetylcholinesterase (AChE) staining, which were not observed in control devices lacking myocytes. Remarkably, nerve electrical recordings progressively improved over 4 weeks, which could be attributed to the biological amplification of the signals and improved tissue integration compared to all-synthetic devices^168^. Biohybrid interfaces with tissue-specific selectivity could be realized by carefully engineering the cell phenotypes in these devices. For instance, myocytes could selectively integrate with motor neurons to restore motor function, sensory neurons could promote and restore sensation, while neuronal or glial cells could facilitate applications in the central nervous system. However, a degree of variability in the extent of integration was observed across different animals in the study, thus raising questions regarding the translatability of such approaches.

#### Biohybrid multielectrode arrays

In the pursuit of effective neural interfaces that seamlessly integrate with host tissue and improve communication with neural circuits, an approach using a ‘biohybrid transition microelectrode array’ has recently been proposed^169^. The device, which looks like a biohybrid equivalent of depth-penetrating MEAs (e.g., the Utah array), consists of a 4 × 4 matrix of pyramidal electrodes that house neural cells. Axons projecting from each electrode into the native tissue are suggested to provide enhanced spatiotemporal resolution compared to conventional MEA implants. While this design aims to facilitate synaptic integration of bioelectronic devices with neural tissues for bidirectional communication (readout and stimulation), to our knowledge the integration and functionality of such devices have not been reported. Further research will be needed to guide axonal projections and synapse formation for high-resolution interfaces.

### Challenges and outlook

Biohybrid interfaces merge cellular constructs with conventional bioelectronics to improve biocompatibility and chronic bio-integration by minimizing FBR and establishing tight coupling with target tissues. Current biohybrid systems rely on existing data acquisition and stimulation systems, which allows them to match the transfer bandwidths and functionalities of biomimetic electronics. However, the translation of biohybrid technologies presents several key challenges. First, the temporal gap between device implantation and the onset of physiologically relevant interactions with host tissues can take several weeks to months^143^, which is a significant hurdle. This delay primarily arises from the time required for the cells within the devices to grow and mature into functional units capable of generating and transmitting electrophysiological signals, as well as for neurite outgrowth and synaptogenesis^143^. Innovations in guided growth, pre-formed axonal tracts, and targeted synaptogenesis present promising avenues for addressing this delay and improving the long-term functionality of biohybrid devices. Moreover, ensuring robust cellular adhesion to the devices is critical not only to prevent detachment during insertion but also to maintain cell retention post-implantation, compromising device functionality, safety, and longevity. Optimizing cell migration and viability, as well as precisely controlling the cell fate if stem cells are employed, are pivotal to ensure targeting specificity and avoid unwanted adverse effects. The material properties of biohybrid devices, including hydrogel swelling, biodegradation, and immunomodulation, must also be finely tuned to ensure long-term stability. The controlled production and quantification of ECM proteins, along with localized delivery of biomolecules and the often-overlooked inclusion of glial cells, could better emulate the natural cellular milieu surrounding biohybrid devices. Other cell types specific to the target application could facilitate synergistic integration with excitable tissues beyond the nervous system, such as skeletal, smooth, or cardiac muscle.

Looking into the future, biohybrid systems hold great potential for advancing regenerative electronics and novel therapeutic interventions across biological scales. Integrating nanomaterials for direct modulation of cellular activity at a cellular level will allow the development of next-generation biohybrid platforms that can be remotely modulated^170^. Such platforms will not only further in vitro studies of cellular communication but will also enable therapeutics for diseases such as visual impairments^171^. On the other hand, by leveraging tissue-engineering approaches, it is possible to design regenerative electronics that can safely integrate with tissues and organs. 3D biohybrid constructs of different geometries have been reported as building blocks of complex tissues. For instance, fiber-like structures of cells and ECM proteins wrapped by a hydrogel shell can assemble into tissues in vitro and form fascicle-inspired 3D tissues like muscle, nerves, and tendons^172,173^. Such structures can form synapses with native tissues and facilitate high-resolution stimulation, which cannot be achieved via conventional deep brain stimulation^174^. Existing biohybrid systems are generally based on passive components with limited adaptability to external cues. Engineering the structural and functional properties of the artificial and living components of biohybrid systems can facilitate the integration of adaptive and the development of intelligent interfaces. For example, a recently reported ferroelectric living interface can facilitate precisely tuned exosome secretion for biomimetic neurovascular remodeling for regenerative medicine and biointegration^175^.

As novel fabrication technologies like 3D bioprinting enable bio-inspired devices with high spatial resolution across multiple scales, the vascularization and innervation of the living components pose significant challenges. Angiogenesis-inspired microfluidic devices^176^ and electrocatalytic on-site oxygenation for cell-laden bioelectronic platforms^177^ are just a few of the strategies that could be adopted to support large biohybrid constructs. Advances in gene editing might also yield interesting developments, including biohybrid engineered cell factories, i.e., implantable bioelectronic devices designed to actively regulate the tissue microenvironment by secreting proteins, neurotransmitters, cytokines, and other biomolecules^178–180^. Research in oxygen generation and immune protection of implanted therapeutic xenotransplants in vivo may enable long-term bioelectronic cell therapies^177,181^. Furthermore, advancements in the in situ assembly of conductive polymers localized extracellularly to living neurons present an exciting avenue for biohybrid device innovation. A general approach for realizing such unique fabrication of substrate-free organic bioelectronics directly in vivo, leverages metabolites present in the tissue for the in situ polymerization of soft conductive gels^182^. Anchoring the conductive polymers to the cell membrane by introducing engineered monomers into the lipid bilayer establishes a close connection between the synthetic materials and cell membrane required for future bioelectronic applications^183^. Alternatively, through genetic modification, specific enzymes can be introduced on cell membranes to catalyze in situ polymerization of conductive polymers for target-specific control over biological interfaces^184^. While biohybrid devices do not often use autologous cells, future iterations could incorporate induced pluripotent stem cells (iPSCs). Future directions for biohybrid devices could further focus on integrating self-healing materials to extend device longevity and developing biosensors for adaptive therapeutic responses, such as controlled compound release or electrical stimulation^185,186^. Embedded cells could act as biological sensors, triggering closed-loop responses to changes in the host microenvironment or device performance^187^. Leveraging biohybrid approaches could lead to multimodal devices combining diagnostic, therapeutic, and regenerative functions within a single, versatile platform.

## Living electronics and interfaces

**Fig. 4:**
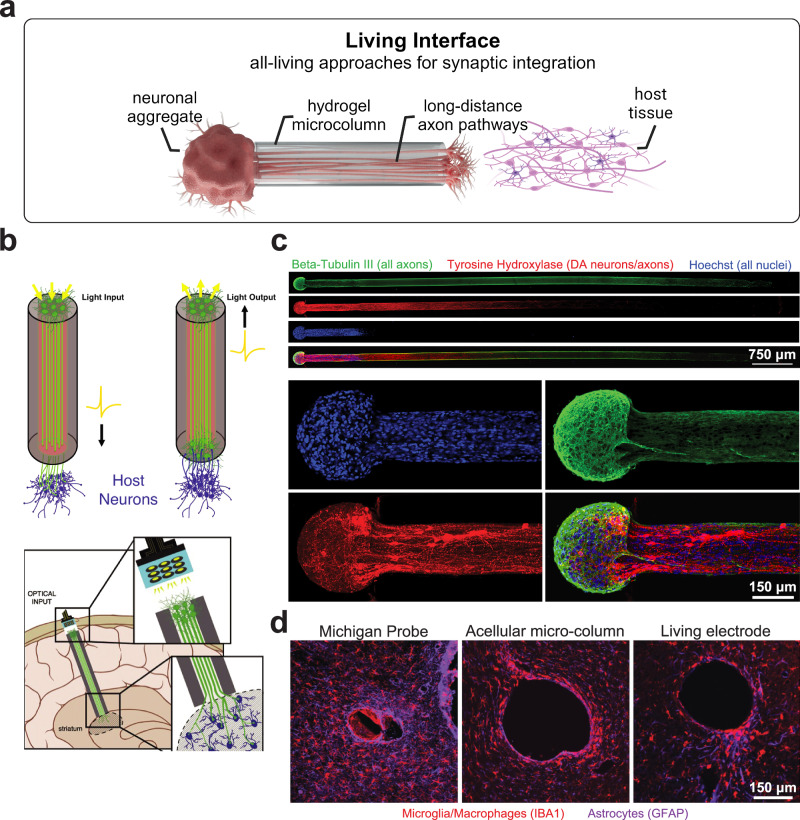
Living interfaces. **a** Schematic of a living electrode, composed of a hydrogel microcolumn seeded with a neuronal aggregate. Long-distance axonal pathways grow along the microcolumn for synaptic integration with the host tissue. **b** μTENNs as a platform technology for bidirectional all-optical living electrodes to record and modulate neural activity^70,188^. **c** Dopaminergic μTENNs for restoration of the nigrostriatal pathway in models of Parkinson’s Disease^191^. **d** Host response at 1-month post-implantation of a Michigan microelectrode, an acellular hydrogel micro-column, and a living electrode, immunolabeled for microglia/macrophages (IBA-1; red) and astrocytes (GFAP; purple)^188^. Panels reproduced with permission from **b**. ref. ^188^., Wiley, and ref. ^70^., Science; **c**. ref. ^191^., Wiley; and **d**. ref. ^188^., Wiley. Panel **a** created with BioRender.com and released under a CC BY-NC-ND 4.0 International license (creativecommons.org/licenses/by-nc-nd/4.0/deed.en).

The term living electronics and interfaces describes systems that are composed exclusively of biologically derived materials and living cells. Compared to biohybrid devices, here living cells do not merely provide a biological interface layer, but instead act as the active input/output terminals within the device. As such, information exchange between the implant and host tissues is primarily recorded, transduced, and modulated by living cells instead of synthetic components^70,188^. This approach could then be leveraged to develop all-living electrodes for bidirectional communication in the central and peripheral nervous systems and in virtually any electrically active tissue in the body. The basic principle involves the use of neuronal axons as signal transducers instead of other conductive materials. The encasement and guidance of such neurons and axonal tracts in a hydrogel microcolumn enable the biofabrication of all-living tissue-engineered medical products ready for implantation (Fig. 4a)^70,188–190^.

In recent years, the concept of living electrodes gained increasing popularity, with applications spanning several neurological conditions^188^. About a decade ago, micro-Tissue-Engineered Neural Networks (μTENNs) were first introduced as methods for fabricating bio-inspired long-distance neuronal pathways^189^. μTENNs consist of a hydrogel microcolumn seeded with a population of aggregated neurons with long-projecting axons growing through the lumen of the microcolumn, giving rise to axon-based living electrodes as a platform technology for communication with the nervous system and restoring lost function after injury or neurodegeneration. While most applications involve single μTENNs^70,188,189,191^, 3D multicellular biocircuits can also be fabricated with nested μTENNs. For example, bidirectional axonal growth of dorsal root ganglion sensory neurons (DRG-SN) may innervate both cortical neurons and cardiomyocytes, showing a proof of concept of fully biological neuromodulatory biocircuits^192^. The functional connectivity of 3D tissue-engineered axonal tracts has also been assessed using calcium fluorescence imaging, highlighting the potential of these constructs as physiologically relevant in vitro platforms for neurological research^193^.

### Tissue engineered axon tracts for synaptic brain-machine interfaces

Tissue-engineered axonal tracts may form a biological link between host and electronics, providing a platform for synaptic-based BMIs. Synaptic-based recording and neuromodulation offer an exquisite combination of specificity and long-term fidelity, potentially enabling prosthetic control, sensory and proprioceptive feedback, as well as stimulation and inhibition of neural circuits^188^. Axon-based living electrodes with multiple neuronal subtypes to stimulate, inhibit, and modulate neural activity have been reported^70,188^. The synaptic integration of single axons with hundreds of host neurons enables high spatial resolution through biological multiplexing, and preferential synaptogenesis based on the neuronal subtypes may result in improved target-specificity^188^. μTENNs with neuronal aggregates on both sides of the microcolumn act as bidirectional living electrodes that provide a biologically-based “all-optical” input/output platform for recording and stimulating the cerebral cortex (Fig. 4b, d)^70,188^. Biofabricated living electrodes with long-projecting glutamatergic axons in hydrogel microcolumns for opto-biological monitoring and modulation of brain activity have been demonstrated, with high specificity and longevity in implantable neural interfaces^70^. Using optogenetic manipulation, light-driven neuromodulation of downstream cortical activity (input) and monitoring of cortical activity (output) may be achieved for targeted readout and control in vivo^70,194^. The survival and integration of a living electrode implanted in a rat model were shown, as well as functional connectivity via intravital calcium imaging with GCaMP-based optical readout following implantation^70^. While these results demonstrated the feasibility of all-living I/O interfaces, the level of synaptic integration and subsequent information transfer bandwidth are remaining challenges. The need for novel stimulation and recording modalities capable of interfacing living electrodes with conventional neuroengineering apparatuses must also be addressed^70^.

Based on the application, living electrodes incorporating various neuronal subtypes have been developed^70,191^. Potential future applications include the treatment of Parkinson’s disease (PD) with ‘living deep brain stimulation’ for dopaminergic regulation (further described in the next section), GABA-based living electrodes to inhibit epileptogenic brain activity in seizures, glutamate-based living electrodes to input sensory feedback from advanced prosthetic limbs, as well as the development of other electroceuticals that modulate neurotransmitter levels or inhibit pathological neural activity^188^.

### Tissue engineered axon tracts for restoring brain circuitry

Tissue engineered axon tracts could also be used as a regenerative strategy capable of rebuilding lost brain circuitry in the adult brain. White matter tracts, those long-projecting axonal pathways that are critical to relay information across brain regions, make up more than half of the human brain volume^195^ but are particularly vulnerable to neurotrauma and neurological disorders^196^. Instead of conventional interconnects and electrodes, implantable micro-tissue-engineered brain pathways contain preformed axons for biological modulation of neural activity, restoring physiological neurotransmitter levels, and replacing lost axonal tracts^191,197–201^. Such living axon-based interfaces may serve as both a biological scaffold for regeneration and active signal transducers that rely on the neurochemically-regulated biofeedback – including synaptic inputs – to appropriately modulate host circuitry. PD is a prime disease model to illustrate the potential of axon-based living electrodes in restoring and treating circuit disorders^202^. PD is a neurodegenerative disease caused by the progressive loss of dopaminergic neurons in the Substantia Nigra Pars Compacta (SNpc) and subsequent denervation and dopamine regulation in the striatum^202^. Neuromodulation devices (e.g., DBS) have been demonstrated as effective therapeutic strategies^203,204^, but they provide symptomatic relief rather than treating the underlying pathological consequences of the disease^205,206^. μTENNs with dopaminergic neurons have been leveraged to create tissue-engineered nigrostriatal pathways (TE-NSPs) that establish dopaminergic inputs to the striatum, demonstrating the survival and synaptic integration of dopaminergic axon-based living tissue with functional dopamine release intrinsically and upon stimulation (Fig. 4c)^191,199,200,202,207^. Multiple lengths of μTENNs for both rat and human scale sizes have been reported, showing the potential of axon-based living tissue for anatomically-appropriate functional restoration of damaged brain circuits^200,201^.

### Living scaffolds for repair and innervation beyond the brain

The use of tissue-engineered axon tracts could also extend beyond the brain and facilitate the regeneration of other excitable issues, including peripheral nerve injury, muscle innervation following volumetric muscle loss, and spinal cord repair. Tissue-engineered nerve grafts (TENGs) have been shown to serve as living scaffolds to accelerate axonal regeneration and functional recovery following peripheral nerve injury^208^. Controlled stretch-growth of axons in mechanobioreactors enables cm-scale aligned axonal tracts that, following implantation to bridge missing segments of peripheral nerve, demonstrate the ability to guide host axon long-distance outgrowth to enable recovery after challenging major peripheral nerve injuries in porcine models^209^. TENGs significantly accelerated regeneration rates compared to nerve guidance tubes and matched those of autografts^208,209^. Similarly, axon regeneration across nerve gaps can extend to spinal cord injury, with living axon-based scaffolds bridging spinal cord injuries and potentially forming local synapses with host axons as functional relays across the lesion^210^. Pre-innervated tissue-engineered muscle, composed of spinal motor neurons and skeletal myocytes on nanofibrous scaffolds, improved regeneration and functional recovery after volumetric muscle loss in a rat model^211^. As these examples show, tissue-engineered living scaffolds with preformed axon tracts can promote host axonal, neuronal, and neuromuscular integration and thus may provide tissue-specific platforms for all-living regenerative electronics that repair, innervate, and modulate host tissue.

### Challenges and outlook

Living electronics and interfaces are composed exclusively of biological components and do not involve any synthetic materials. Therefore, they do not utilize conventional systems for recording and stimulating electrophysiological activity. Living electrodes and interfaces generally rely on optical imaging^70^ and remote stimulation paradigms^212,213^ for recording and stimulation, respectively. This reliance on fluorescence microscopy limits the transfer bandwidth, given the low temporal resolution of current imaging systems and fluorescent reporters. Advances in ultra-fast fluorescence microscopy will directly enhance the capabilities of current living interfaces.

To successfully transition all-living interfaces from laboratory settings to the clinic, chronically stable structural and functional integration with host tissues is critical. Specifically, promoting tissue regeneration, preventing fibrosis or rejection, and adapting to individual patient anatomy and pathology are key areas for future improvement. Variability between in vivo animal studies and reliance primarily on post-mortem histological analysis to confirm appropriate synaptic integration might delay the technology development. These issues may be mitigated by integrating non-invasive monitoring and diagnostic capabilities into the devices to facilitate tracking and adaptation of their performance and tissue response over time. The information transfer bandwidth of synaptic-mediated living electronics is intrinsically limited by the number of synapses at the tissue/device interface and decoding the output neuronal activity is a significant challenge. However, the input mechanisms are more straightforward and provide a targeted, specific neurotransmitter replacement strategy that is inherently self-renewing. Strategies for guided axonal outgrowth could improve viability and integration post-transplantation, while cell-specific control and targeted synaptogenesis could enable biologically mediated interface selectivity and precise therapeutic interventions. This strategy essentially mitigates a chronic FBR^70,188,207^; however, it presents other unique challenges, including significant regulatory hurdles, as the successful clinical translation further requires scalable and reproducible manufacturing for patient-specific devices, with consistent survival, outgrowth, synaptogenesis, and chronic functional stability.

Non-invasive methods for monitoring and modulating living interfaces are also crucial^212,213^. Mesh nanoelectronics have already been integrated with organoids without disrupting their growth, offering chronic tissue-wide electrophysiology with high spatiotemporal resolution^214^. Interfacing nanomaterials with neuronal aggregates could leverage optical, magnetic, electrical, and thermal means for non-invasive neuromodulation of ‘cyborg’ living electrodes. For instance, photothermally active nanomaterials interfaced with neuronal aggregates can alter the electrophysiology of living electrodes for non-invasive, all-optical, and non-genetic neuromodulation^212^. Minimally invasive removal of devices or their non-viable components is another critical area of focus. Strategies may include optical stimulation for reversible activation or deactivation, pharmacological interventions, or a built-in genetic ‘kill-switch’ that ensures patient safety^215,216^. Precisely targeting the desired neuronal subpopulation(s) for appropriate circuit-level modulation is another challenge, with advances in neuronal differentiation and gene editing offering new possibilities for tuning synaptic integration to enable living electrodes that inhibit, excite, or modulate activity based on the specific application. Synthetic biology toolkits, including gene editing of living cells and viruses, are also already increasingly used as living building blocks for electronics, sensing components, or power sources, giving rise to the field of ‘living synthelectronics’^217^.

### Conclusion

Neural interfaces and electronics are progressing toward bio-inspired designs through careful engineering of device materials and architecture. These approaches allow artificial neural technologies to mimic native biological tissues and mitigate the detrimental FBR. Strategies for realizing bio-inspired designs can be classified into biomimetic, bioactive, biohybrid, and living interfaces. In this review, we have discussed the fundamental design principles behind each of the bio-inspired device strategies and summarized the related seminal device constructs (Table 1). The discussed bio-inspired platforms establish bi-directional communication with target neural tissues and also provide a platform for regenerative tissue engineering.

**Table 1:** Summary of the design principles, key properties, advantages, current limitations, and status of clinical translation for each of the bio-inspired electronics strategies

	Biomimetic electronics	Bioactive electronics	Biohybrid electronics	Living interfaces
**Design principles**	Optimized device geometry (thin films^52–55^, flexible threads^43,48–51^, mesh electronics^45–47^); Soft materials (polymers^78^, hydrogels^3,58^, elastomers^56^)	Bioactive coatings composed of adhesion molecules^113^, ECM proteins^115^, antibodies^116^, peptides^117^.	Biological coating of cellular constructs cells^68^.	Composed exclusively of biological components and cells^191^.
**Mechanical properties**	Flexible interfaces with higher Young’s modulus ($E \sim 10-{10}^{6}{kPa}$); Moderate compliance with target tissues.	Similar properties as biomimetic electronics ($E \sim 10-{10}^{6}{kPa}$); Moderate compliance with target tissues.	Softer interfaces ($E \sim 10-{10}^{4}{kPa}$); Better compliance with target tissues.	Similar mechanical properties as biological systems ($E \sim 1-{10}^{3}{kPa}$); Highest compliance with target tissues.
**Electrical properties**	High electrical conductivity ($\sigma \sim {{10}^{3}-10}^{8}{S}/m$); High electrochemical capacitance.	Electrical and electrochemical properties affected by bioactive coatings ($\sigma \sim {{10}^{2}-10}^{8}{S}/m$).	Electrical and electrochemical properties affected by cellular coatings ($\sigma \sim {{10}^{2}-10}^{5}{S}/m$).	Similar to native cells and tissues.
**Transfer Bandwidth**	High	High	High	Low
**Advantages**	Established fabrication; Safe materials; Compatible with existing recording and stimulation systems.	High biocompatibility; Low FBR; Compatible with existing recording and stimulation systems.	High biocompatibility; Low FBR; Potential for single-cell resolution; Compatible with existing recording and stimulation systems.	Highest biocompatibility; Minimal FBR; Excellent synaptic integration with host tissue.
**Current Limitations**	FBR may result in device encapsulation and limited longevity in vivo; Limited spatial resolution.	Limited stability of bioactive coatings; Limited spatial resolution.	Complex fabrication; Technical challenges in maintaining cellular constructs; Limited chronic functionality.	Complex fabrication; Limited structural and functional stability; Reliance on imaging modalities.
**Status of Clinical Translation**	Under clinical translation^74–76^	Pre-clinical validation^65,114,119^	Experimental; Short-term pre-clinical studies^144,194^	Early-stage research in animal models^191^.

Several regulatory, technical, and biological hurdles must be addressed before these technologies can achieve widespread clinical application. A key challenge lies in defining the precise interactions and mechanisms of action of the devices and meeting the rigorous safety and efficacy standards required by agencies such as the FDA and EMA. From a technical standpoint, the devices should exhibit chronic structural and functional stability while minimizing host immune response at the tissue-device interface. Biomimetic devices such as neural threads (Neuralink)^75^, stentrodes (Synchron)^74^, and thin-film microECoG grids (Precision Neuroscience)^76^, have already received breakthrough device designation from the FDA to speed up their development and regulatory process. This has been achieved largely due to the substantial research efforts and investments in biomimetic devices, as well as their reliance on well-established manufacturing processes and materials approved for clinical use. Recently, Science Corporation achieved a significant milestone in validating biohybrid electronics by integrating them with existing cortical structures^194^. This proof-of-concept demonstration paves the way for the future development and translation of high-bandwidth BCIs to guide goal-directed behavior.

Conjugating biologically derived materials such as proteins and cells to electronic platforms increases device complexity and considerations for clinical translation. While regulatory pathways for tissue-engineered products, such as cell-based therapies^218,219^, provide some guidance, they remain underdeveloped for devices that integrate biological and electronic components. Here, it is crucial to establish the bio-integration and chronic stability of all interfaces. For cell-containing and all-living devices, it is essential to ensure precise control over cell fate, migration, and integration. Furthermore, secondary mechanisms of action, such as neurotransmitter release or remodeling of the interface’s microenvironment, must be fine-tuned to prevent off-target effects.

Another critical consideration for the eventual clinical translation of these emerging technologies is to scale up manufacturing while following current Good Manufacturing Practices (cGMPs)^220^. Although cGMPs add complexity to the fabrication schemes, require tightly controlled culture conditions, and mandate reproducibility and reliability of laboratory research for effective clinical applications, they ensure that the developed medical devices meet all applicable requirements and specifications for safe operation. As the distinction between living and synthetic components gets increasingly blurred, it is imperative to navigate the complex network of technical, ethical, and regulatory considerations for the responsible development of next-generation bio-inspired neural interfaces that are safe, effective, equitable, and accessible to patients, regardless of their geographical and socio-economic status.

### Reporting summary

Further information on research design is available in the Nature Portfolio Reporting Summary linked to this article.

## Supplementary information

Reporting Summary
